# Adherence to Labor Arrest and Failed Induction of Labor Guidelines: The Impact of a Quality-Improvement Educational Intervention

**DOI:** 10.3390/jcm13164720

**Published:** 2024-08-12

**Authors:** Jennifer J. M. Cate, Christopher K. Arkfeld, Meagan Campol, Katherine H. Campbell, Christian M. Pettker, Jessica L. Illuzzi

**Affiliations:** 1 Department of Obstetrics, Gynecology and Reproductive Sciences, Yale School of Medicine, New Haven, CT 06510, USA; 2 Section of Maternal-Fetal Medicine, Department of Obstetrics and Gynecology, Duke University School of Medicine, Durham, NC 27710, USA; 3 Section of Maternal-Fetal Medicine, Department of Obstetrics, Gynecology and Reproductive Sciences, Yale School of Medicine, New Haven, CT 06510, USA; katherine.campbell@yale.edu (K.H.C.);

**Keywords:** adherence to labor arrest criteria, cesarean reduction, cesarean delivery, educational intervention, labor arrest disorder, labor arrest adherence, quality improvement

## Abstract

**Background/Objective:** To evaluate adherence to labor arrest and failed induction of labor (IOL) criteria in nulliparous, term, singleton, and vertex (NTSV) cesarean deliveries at an academic medical center and to measure the impact of a quality-improvement educational initiative that focused on obstetric provider education of modern labor arrest and failed IOL criteria. **Methods:** This is a retrospective cohort study using electronic health record (EHR) data with a pre- (1 September 2018–30 September 2019) and post-intervention (1 October 2019–31 March 2020) study design of all NTSV cesarean deliveries for labor arrest or failed IOL performed at an academic medical center in the northeastern United States. The quality-improvement educational intervention consisted of the distribution of educational pocket cards outlining modern labor arrest and failed IOL criteria to obstetric providers. Outcomes included adherence to labor arrest and failed IOL criteria pre- and post-intervention with secondary outcomes evaluating adherence by provider type (Maternal–Fetal Medicine (MFM) or generalist obstetrician). Descriptive and bivariate statistics were used in the analysis. **Results:** Pre-intervention, 272 NTSV cesarean deliveries were performed for labor arrest or failed IOL versus 92 post-intervention. Adherence improved post-intervention amongst failed IOL (OR 6.5, CI 1.8–23.8), first-stage arrest (OR 4.5, CI 2.2–10.8) and second-stage arrest (OR 3.7, CI 1.5–9.4). When comparing provider type, MFM physicians were more likely to be adherent to labor arrest and failed IOL criteria compared to generalist obstetricians pre-intervention (OR 3.1, CI 1.7–5.5); however, post-intervention, there was no longer a difference in adherence (OR 3.3, CI 0.9–12.3). **Conclusions:** Adherence to labor arrest criteria was suboptimal in the pre-intervention period; however, a targeted quality-improvement educational intervention improved adherence rates to labor arrest and failed IOL criteria among obstetric providers.

## 1. Introduction

The cesarean delivery rate has increased from 1996 onward, peaking in 2009 at 32.9% [1]. Despite a temporary period of decline from 2013 to 2016, in 2017, the cesarean delivery rate increased to 32% and has remained consistent since, with the most recent rate in 2022 at 32.2% [2]. Although cesarean deliveries are often performed out of concern for fetal or maternal status, the markedly increased cesarean delivery rate has not been associated with significant improvements in outcomes [3], with concern for worsening maternal morbidity [4].

In a population-based retrospective cohort study between 2003 and 2009, Barber et al. reviewed the indications for cesarean delivery to evaluate the potential etiology of the rising cesarean delivery rate. Their findings demonstrated a 50% increase in primary cesarean deliveries with 34% of that increase related to labor arrest [5]. In 2010, Zhang et al. evaluated contemporary labor data in order to create a modern labor curve, redefining active labor as beginning at 6 cm while allowing longer durations in the latent phase of labor [6]. This study helped to establish the basis of the 2012 *Summary of the Society for Maternal–Fetal Medicine*, *Eunice Kennedy Shriver National Institute of Child Health and Human Development*, *and American College of Obstetricians and Gynecologists Workshop*, *Preventing the First Cesarean Delivery*, which recommended modern labor arrest criteria encouraging additional time prior to diagnosing labor arrest and failed induction [7]. Cross et al. evaluated the cesarean delivery rate from 2009 to 2013 to assess the temporal relationship of cesarean delivery rates and indications with these studies and consensus guidelines [8]. They noted that the cesarean rate in the study population decreased from 36.5% to 31.4%, with 74% of the decrease secondary to fewer primary cesarean deliveries, which was largely attributed to a reduction in cesarean deliveries performed for labor arrest [8]. This study demonstrated the potentially modifiable proportion of the cesarean delivery rate that could be impacted by improved adherence to labor arrest criteria.

Despite the potential opportunity to improve the cesarean delivery rate by following these guidelines, concern over lack of adherence remains. Multiple studies highlight that the majority of cesarean deliveries do not meet consensus guidelines [9,10]. Given these findings, initiatives to improve adherence to modern labor guidelines are of paramount importance in reducing potentially unnecessary cesarean deliveries; however, the literature describing evidence-based approaches to achieve this goal is scarce. The objective of this study was to evaluate baseline adherence to labor arrest and failed induction of labor (IOL) criteria in nulliparous, term, singleton, and vertex (NTSV) cesarean deliveries at a tertiary academic medical center in the northeastern United States and to measure the impact of a quality-improvement educational intervention on subsequent labor arrest adherence rates.

## 2. Materials and Methods

### 2.1. Study Design

This is a retrospective cohort study using the electronic health record (EHR) with a pre- (1 September 2018–30 September 2019) and post-quality-improvement intervention (1 October 2019–31 March 2020) study design on all NTSV cesarean deliveries performed at a tertiary care center in the northeastern United States. The study period was truncated to March of 2020 given the start of the COVID-19 pandemic and the significant changes in clinical practice that ensued. Individuals were included if nulliparous and at term gestational age (at least 37 weeks gestation) at the time of delivery with a singleton pregnancy in vertex presentation who underwent cesarean delivery for labor arrest or failed IOL during the aforementioned time periods. Exclusion criteria included preterm delivery, multifetal gestation, fetal malpresentation, intrauterine fetal demise, history of prior cesarean delivery, indication of cesarean delivery outside of labor arrest, or failed IOL or multiple indications for cesarean delivery.

For context, our labor and delivery (L&D) unit is staffed by resident physicians and midwives, in addition to supervising the Maternal–Fetal Medicine (MFM) faculty, and generalist obstetricians who are affiliated with our institution but remain in the private sector. Two thirds of patients delivering at our institution are private patients of the generalist obstetricians with the other one third of patients (high-risk and university patients) cared for by the MFM faculty. This study was considered exempt by our institutional IRB.

### 2.2. The Intervention

**Figure 1:**
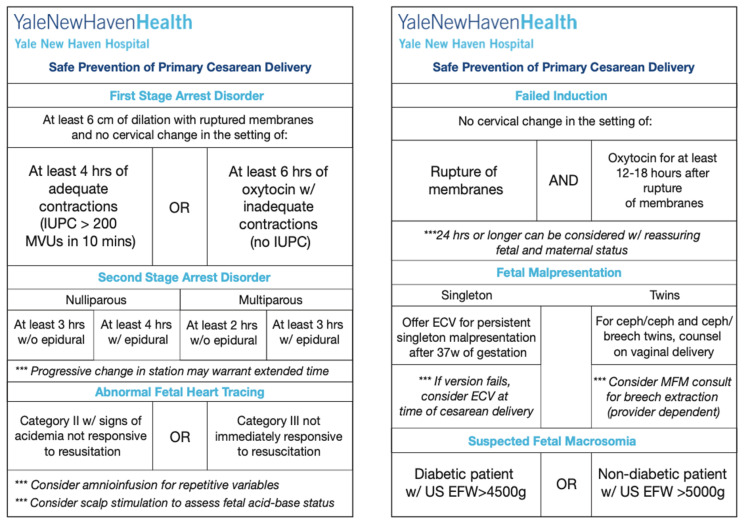
Pocket cards developed through a quality-improvement project to educate providers on labor arrest criteria.

In order to create a structure for institutional quality-improvement interventions aimed at safely reducing cesarean delivery rates, a multi-disciplinary group entitled the Committee to Support Vaginal Birth (CSVB) was formed. This group consisted of volunteer labor and delivery nurses, midwives, resident physicians, generalist obstetricians, MFM fellows and attendings and anesthesia colleagues. All interested members were welcomed into the committee, which convened monthly beginning in June 2019. Based on studies suggesting a gap in provider knowledge surrounding modern labor arrest criteria [9,10], the CSVB devised an intervention to educate obstetric providers at our institution on modern criteria for labor arrest and failed IOL based on the 2014 ACOG and SMFM recommendations in the *Safe Prevention of the Primary Cesarean* [11]. The initial quality-improvement intervention consisted of designing and distributing an educational pocket card summarizing the criteria for labor arrest and failed IOL (Figure 1). The compact design of the pocket card provided ease of use and proximity to clinical care. Of note, cesarean indications outside of labor arrest were also listed on the card in anticipation of future potential CSVB initiatives not addressed in this intervention. Multiple iterations were evaluated by both the CSVB as well as the departmental Patient Safety and Quality Improvement Committee with agreement by these committees on language and content. The pocket cards were printed and made widely available to all labor and delivery staff by departmental leadership starting on 1 October 2019. Distribution occurred at recurring obstetric huddles, across nursing charting areas, in triage and community offices. At the time of distribution, a review of labor arrest and failed induction of labor guidelines utilizing the pocket card as well as discussion for inter-disciplinary use of the pocket cards was performed. An electronic quick response (QR) code linking to a PDF of the card was placed in convenient clinical areas for additional reference. Furthermore, enlarged posters with similar but expanded information were placed in provider common areas for review (Supplementary Materials). Importantly, no other interventions which aimed to improve labor arrest or failed IOL adherence were pursued during the study period in order to prevent potential confounding from multiple interventions.

### 2.3. Data Abstraction

Individual patient electronic health records (EHRs) were carefully reviewed by trained study team members for the following information: age, parity, gestational age at delivery, labor progress and interventions, primary indication for cesarean delivery and delivering provider. The primary indication for cesarean delivery in addition to delivering provider was obtained from the operative record within the EHR. As mentioned, cases with multiple indications or an indication outside of labor arrest or failed IOL were not included.

Detailed labor data, including cervical dilation and timing of examination, oxytocin administration, timing of membrane rupture including artificial rupture and use of intrauterine pressure catheters, were abstracted. This information was used to assess whether individual patients met criteria for labor arrest or failed IOL as recorded in the operative report. The criteria for labor arrest and failed IOL were defined based on the 2014 ACOG and SMFM recommendations in the *Safe Prevention of the Primary Cesarean Delivery* [11]. For the purpose of our study, we assessed if minimum criteria for labor arrest or failed induction of labor were met. For individuals diagnosed with failed induction of labor, labor data were assessed to determine if they remained less than 6 cm dilated with administration of oxytocin for at least 12 h after membrane rupture and absence of active labor. Of note, our institution encourages exceeding the minimum 12–18 h and supports up to 24 h with reassuring maternal or neonatal status. In individuals with first-stage arrest, labor data were assessed to identify if the cervical dilation was at least 6 cm dilated with a minimum of 4 h elapsed after rupture of membranes with adequate contractions based on an intrauterine pressure catheter and calculated Montevideo units or 6 h of oxytocin administration after rupture of membranes in the absence of adequate contractions. Second-stage arrest was determined by calculating elapsed time from initiation of pushing to the time at which cesarean delivery was recommended to determine if 3 h without epidural or 4 h with epidural had elapsed in these nulliparous patients. The initiation of pushing was chosen rather than time at full dilation, given that the recommendation at the time of the study incorporated the total time of pushing in contrast to total time in the second stage. In situations with a documented prolonged pause in pushing, the pause interval was not incorporated into the total time of propulsive efforts. In rare circumstances where a cesarean decision time was not documented, the cesarean incision start time was used as a surrogate for determining elapsed time.

### 2.4. Outcomes and Analysis

The primary outcomes were adherence to failed induction of labor, first-stage arrest, and second-stage arrest, compared pre- and post-intervention. Secondary outcomes included adherence to labor arrest or failed induction criteria by provider type (MFM faculty versus generalist obstetrician), pre- and post-intervention. Adherence was calculated through the proportion of cesarean deliveries for each individual category (failed IOL, first- or second-stage arrest) meeting criteria as noted above amongst total cesarean deliveries performed for that indication. Bivariate statistical analysis compared adherence rates across intervention periods. Run charts were used to demonstrate adherence and NTSV cesarean delivery rates over time.

## 3. Results

From 1 September 2018 to 30 September 2019, 1506 cesarean deliveries were performed, of which 519 (34.4%) were categorized as NTSV cesarean deliveries. MFM physicians performed 146 (28.1%) NTSV cesarean deliveries with 373 (71.8%) performed by generalist obstetricians. Of the NTSV cesarean deliveries, a total of 272 (52%) were performed solely for labor arrest indications: 95 (34.9%) for first-stage arrest, 111 (40.8%) for second-stage arrest and 66 (24.2%) for failed IOL. Of those, 28.4% (n = 27) met criteria for first-stage arrest, 45.9% (n = 51) met criteria for second-stage arrest and 53.0% (n = 35) met criteria for failed IOL. At baseline, generalist obstetricians adhered to labor arrest guidelines in 35.0% (73/208) of patients versus 61.9% (40/64) in MFM providers.

After initiation of the educational intervention from 1 October 2019 to 31 March 2020, there were 802 cesarean deliveries performed, of which 209 (26.0%) were categorized as NTSV cesarean deliveries. During this period, MFM physicians performed 60 (28.7%) of the 209 NTSV cesarean deliveries with generalist obstetricians performing 149 (71.2%). Of the NTSV cesarean deliveries, 92 (44.0%) were performed solely for labor arrest: 38 were for first-stage arrest with 65.7% adherence (n = 25), 29 for second-stage arrest with 75.8% adherence (n = 22), and 25 for failed IOL with 88% adherence (n = 22). Generalist obstetricians adhered to labor arrest criteria 69.6% (46/66) of the time compared to 88.4% (23/26) among MFM physicians post-intervention.

Odds ratios (ORs) of adherence to labor arrest and failed induction of labor criteria before and after the intervention were calculated by provider type and labor arrest category (Table 1). After the educational intervention, all obstetric providers were 4.2× [CI 2.4–7.1] more likely to adhere to labor arrest criteria. When comparing provider type, MFM physicians were more likely to adhere to labor arrest criteria (OR 3.1, CI 1.7–5.5) compared to generalist obstetricians pre-intervention; however, post-intervention, there was no longer a statistical difference in adherence between MFM providers and generalist obstetricians. When comparing MFM provider adherence pre- and post-intervention, there were no differences in adherence across the intervention [OR 1.4, CI 0.7–2.8]. Generalist obstetricians demonstrated a significant improvement post-intervention compared to pre-intervention with generalist obstetricians more likely to adhere to the guidelines after intervention [OR 1.9, CI 1.3–3.1].

**Figure 2:**
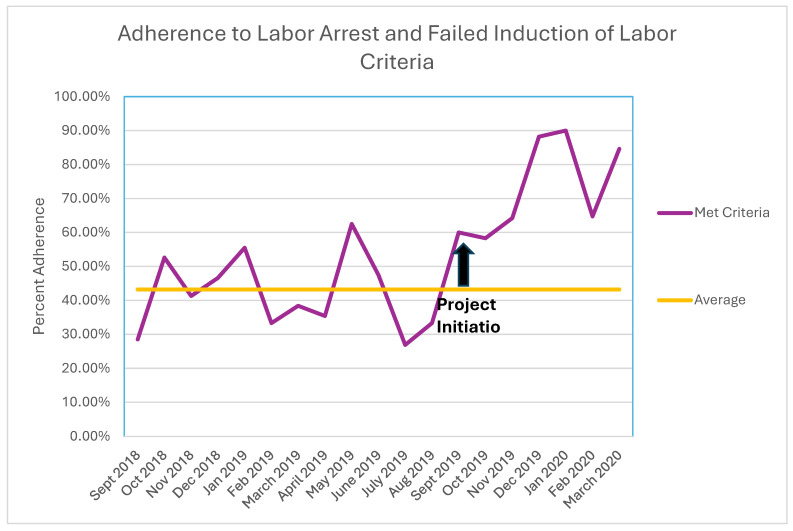
Run chart demonstrating overall adherence to labor arrest and failed induction of labor criteria over time.

The adherence rates for each category before and after the intervention are demonstrated in a run chart (Figure 2). When comparing by arrest types, all categories including failed IOL (OR 6.5 [CI 1.8–23.8]), first-stage arrest (OR 4.5 [CI 2.2–10.8]) and second-stage arrest (OR 3.7 [CI 1.5–9.4]) demonstrated improved adherence post-intervention compared to pre-intervention.

**Figure 3:**
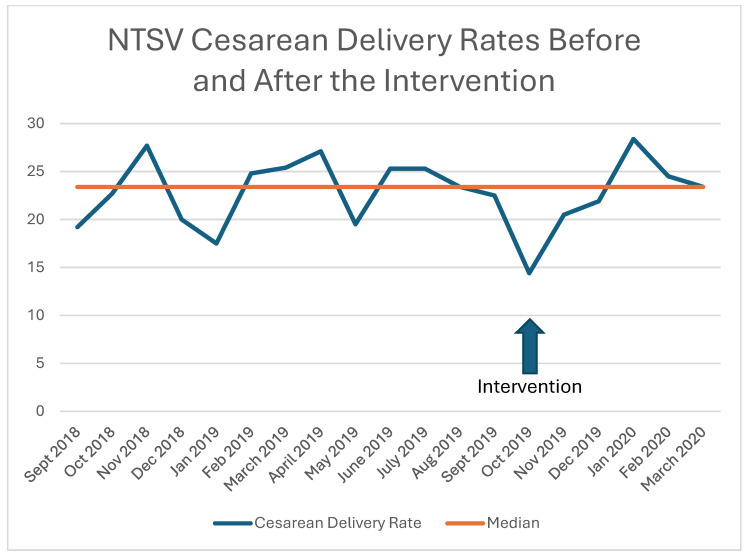
Run chart demonstrating NTSV cesarean delivery rates before and after the intervention.

Additionally, NTSV cesarean delivery rates were calculated before and after the intervention on a monthly basis and demonstrated in a run chart (Figure 3).

## 4. Discussion

This study describes the multi-disciplinary development of an educational intervention to address obstetric provider and staff education of labor arrest and failed IOL guidelines and its impact on obstetric provider adherence to those guidelines. Our study demonstrated that overall baseline adherence to the modern labor arrest criteria at our institution was sub-optimal, with the majority of cesarean deliveries in the pre-intervention period not meeting current consensus guidelines. We subsequently found that implementation of the quality-improvement educational intervention resulted in a significant improvement in adherence to labor arrest and failed induction of labor criteria. In the pre-intervention period, MFM providers were found to be significantly more likely to be adherent to labor arrest and failed induction of labor guidelines than their generalist obstetrician counterparts. Post-intervention, there was an increase in adherence among generalist providers. Additionally, there was no longer a difference in adherence between generalist obstetricians and MFM providers post-intervention. Our findings demonstrate that an educational intervention is associated with improvement in adherence and suggests that a focus on education may remove some barriers to following modern labor arrest and failed induction of labor guidelines. Differential support in ongoing education and review of the current literature may underscore the differences appreciated between the academic and private faculty.

Multiple studies are consistent with our findings, demonstrating that the majority of cesarean deliveries do not meet consensus guidelines for labor arrest [9,10,12,13]. We noted significant baseline differences in pre-intervention adherence between academic MFM providers and generalist obstetricians. This is consistent with a prior retrospective cohort study by Alrais et al. demonstrating that 73% of individuals were non-adherent to guidelines in term, live, and singleton cesarean deliveries [9]. Though directly attributing the difference in practice patterns remains outside of the scope of the current study, given our results, a lack of education may place a role in these underlying differences between the academic MFM providers and the generalist providers based in the private sector. These findings are consistent with Escobar et al., who also demonstrated in their retrospective review that the majority of cesarean deliveries at their institution did not meet criteria of the consensus guidelines, including 69.0% of the cesarean deliveries for failed IOL and 55.4% for arrest of descent, consistent with our study results [10]. Though the *Safe Prevention of the Primary Cesarean Delivery* [11] was published in 2014 suggesting adjustment of labor arrest and failed induction of labor criteria, our study supports the existing literature that demonstrates that these guidelines have yet to be robustly adapted, even at academic centers with an imbalance between private generalist and academic provider adherence. A quality-improvement study by Telfer et al. addressed implementation of an evidence-based bundle including an early labor triage guide, labor walking path, partograph, and pre-cesarean checklist to reduce early labor admissions and adherence to labor arrest guidelines, which demonstrated that knowledge of labor management amongst obstetric personnel increased from 35% to 100% in regard to labor arrest [14]. Our study further adds to the existing literature that education likely remains a necessary component to improve adherence to modern guidelines and quality-improvement educational interventions can likely play a key role in administering this education. Despite the intervention, adherence still ranged from 65.7 to 88%, which may be further optimized with additional interventions such as pursuit of alternative educational interventions, provider feedback of adherence rates as well as cesarean delivery rates, and pre-cesarean delivery decision huddles.

Adherence to modern labor arrest criteria has been shown to have a mixed impact on cesarean delivery rates [13,15,16]. Greenberg et al. demonstrated that the primary cesarean rate decreased with increased adherence to labor arrest and failed induction criteria based on the 2012 recommendations from “*Preventing the First Cesarean Delivery*” [7]. Alternatively, Rosenbloom et al. showed in their retrospective study of a prospective cohort of low-risk individuals that the cesarean delivery rate did not change between 2010 and 2014 after the relaxation of arrest and failed induction of labor guidelines [16]; however, this study did not evaluate adherence to those guidelines. Historically, quality-improvement methods have been shown to be successful in reducing cesarean deliveries. Chaillet et al. demonstrated in a cluster-randomized trial that implementation of best practices in conjunction with cesarean delivery audits and physician feedback yielded a small but statistically significant impact on their cesarean delivery rate, specifically in low-risk populations after stratification [17]. Vadnais et al. targeted provider education and feedback as well as implementation of several policies supporting labor, which cumulatively decreased their NTSV cesarean delivery rate [18]. In a Cochran review by Chen et al., three studies targeting healthcare professionals including implementation of guidelines with a mandatory second opinion, implementation of guidelines with audit and feedback as well as physician education by a local opinion leader demonstrated a reduction in cesarean deliveries [19]. These studies highlight the potential positive impact that quality-improvement interventions can have on cesarean delivery rates. Although our study did not specifically aim to evaluate the impact of the intervention on cesarean delivery rates, this is an important consideration. For instance, there is a potential that part of the providers’ improved adherence was related to improved documentation or classification of indication for cesarean delivery with a reduction in the proportion of cesareans performed solely for labor arrest or failed induction of labor without change in the rate of cesarean deliveries. Given the limited resources that emerged during the COVID-19 pandemic, this evaluation of cesarean deliveries was unable to be performed but is a valid consideration in a study like this and would benefit from further exploration.

The strengths of our study include the development of a quality-improvement educational intervention with various stakeholders including nursing staff, resident and fellow physicians, midwives and attending providers. These multi-disciplinary efforts likely contributed to the intervention’s success. Additionally, our simple educational intervention with pocket cards, QR codes and expanded posters is cost-effective and could be adapted in a variety of different practice settings with varying resources. Methodically, individual patient charts underwent a comprehensive review by experienced study personnel for detailed, accurate information regarding the individual patient labor course and cesarean delivery in order to ensure internal validity. Additionally, no other interventions were pursued to improve labor arrest, failed induction of labor adherence or cesarean delivery rates during the study period, limiting confounding.

Our study should be considered in light of the following limitations. Although we found poor baseline adherence to labor arrest criteria, our study did not evaluate the underlying causality of why providers were not following current guidelines. This is likely a multi-faceted problem and not limited to the education of providers alone. Alternatively, patient and provider demographics and practice setup at our single academic institution may not be generalizable to all populations or clinical settings, thus limiting external validity. Though only one intervention was introduced during our study period, there may be an unmeasured positive effect on adherence rates after the formation of the *Committee to Support Vaginal Birth*. We did not perform direct evaluation of provider knowledge of labor arrest and failed induction of labor criteria before and after the educational intervention, which would have been an informative addition to this study. Furthermore, our study evaluated NTSV cesarean delivery rates before and after the intervention but was unable to evaluate important balancing measures including adverse maternal and neonatal outcomes. Some studies have demonstrated that improved adherence is not at the expense of these [9,10,15]. ACOG with the support of SMFM released a clinical practice guideline entitled “*First and Second Stage Labor Management*” in January 2024 that highlighted changes in evidence-based labor management as well as diagnosis of arrest disorders, specifically second-stage arrest [20]. Our study does not incorporate the most recent guidelines with results that should be considered in this context. Lastly, because of the timing of the COVID-19 pandemic and redistribution of resources, additional audit and plan–do–study–act cycles were unable to be accomplished given the limited resources available to complete these tasks.

In summary, the adherence to criteria for labor arrest disorders was suboptimal in the pre-intervention period. Our targeted quality-improvement initiative significantly impacted adherence rates to labor arrest criteria prior to performing cesarean delivery. Future studies evaluating the implementation of this quality-improvement educational initiative at alternative sites should be considered to continue to evaluate the utility and impact at alternative clinical sites. Additionally, this educational initiative could be considered as a decision aid for alternative indications for cesarean delivery, such as the evaluation and management of intrapartum fetal distress. New guidelines, such as those recently published by ACOG, could be easily adapted and studied to assess the impact of those guidelines on adherence and cesarean delivery rates. Lastly, studies aimed at improving adherence to labor arrest criteria should be pursued in order to optimize cesarean delivery rates for this common indication and avoid potentially unnecessary cesarean deliveries.

## Figures and Tables

**Table 1:** Comparison of adherence to labor arrest and failed induction of labor criteria by provider type and diagnosis.

	Pre-Intervention (n, %) Total Cesarean Deliveries (n = 1506)	Post-Intervention (n, %) Total Cesarean Deliveries (n = 802)	Odds Ratio [CI]
NTSV Cesarean Deliveries	519 (34.4%)	209 (26.0%)	
Labor arrest or Failed IOL	272 (52.4%)	92 (44.0%)	
Failed Induction of Labor	95 (34.9%)	38 (41.3%)	
First-Stage Arrest	111 (40.8%)	29 (31.5%)	
Second-Stage Arrest	66 (24.2%)	25 (27.1%)	
Adherence by Arrest or Failed IOL			
Failed IOL	35/66 (53.0%)	22/25 (88.0%)	6.5 [1.7–23.8]
First-Stage Arrest	27/95 (28.4%)	25/38 (65.7%)	4.5 [2.2–10.8]
Second-Stage Arrest	51/111 (45.9%)	22/29 (75.8%)	3.7 [1.5–9.4]
Adherence by Provider Type			
MFM	40/64 (62.5%)	23/26 (88.4%)	1.4 [CI 0.7–2.8]
Generalist Obstetrician	73/208 (35.1%)	46/66 (69.6%)	1.9 [CI 1.3–3.1]

## Data Availability

The datasets presented in this article are not readily available secondary to technical and logistical constraints. Requests to access the datasets should be directed to Jennifer Cate.

## Supplementary Materials

The following supporting information can be downloaded at https://www.mdpi.com/article/10.3390/jcm13164720/s1. Figure S1: Expanded Recommendations for the Safe Prevention of the Primary Cesarean Delivery.
