# Supplementary material for: Adherence to Labor Arrest and Failed Induction of Labor Guidelines: The Impact of a Quality-Improvement Educational Intervention
Source: J Clin Med. 2024 Aug 12;13(16):4720. doi: 10.3390/jcm13164720 (PMC11355156; doi:10.3390/jcm13164720)
Supplement: Supplementary file 1 [file jcm-13-04720-s001.zip › jcm-3102079-supplementary.pdf]

YaleNewHaven**Health**

Yale New Haven Hospital

Recommendations for the Safe Prevention of Primary Cesarean Delivery

Failed Induction

No cervical change in the setting of:	Rupture of membranes<br>AND<br>oxytocin for at least 12-18 hours after rupture of membranes	*** Cervical Ripening should be used in the setting of an unfavorable cervix	*** 24 hours or longer after rupture of membranes can be considered w/ reassuring fetal and maternal status

First Stage Arrest Disorder

At least 6 cm of dilation with ruptured membranes and no cervical change in the setting of:	At least 4 hrs of adequate contractions (IUPC w/ >200 MVUs in 10 min)<br>OR<br>At least 6 hrs of oxytocin administration w/ inadequate contractions (or no IUPC)	*** A prolonged latent phase (<6 cm dilation) should not be an indication for cesarean delivery	*** Consider oxytocin, fluid and glucose needs

Second Stage Arrest Disorder

Nulliparous	Multiparous			
At least 3 hrs w/o epidural	At least 2 hrs w/o epidural	*** Progressive change in station may warrant extended time on an individualized basis	*** Operative vaginal delivery in the second stage of labor by experienced and well trained physicians should be considered a safe, acceptable alternative to CD	*** Manual rotation of the fetal occiput in the setting of fetal malposition in the second stage of labor is a reasonable intervention to consider before moving to operative vaginal delivery or CD
At least 4 hrs w/ epidural	At least 3 hrs w/ epidural			

Abnormal Fetal Heart Tracing

Category II w/ signs of acidemia not responsive to resuscitation	OR	Category III not immediately responsive to resuscitation

***Amnioinfusion for repetitive variable fetal heart rate decelerations may safely reduce the rate of cesarean delivery

***Scalp stimulation can be used as a means of assessing fetal acid-base status when abnormal or indeterminate fetal heart patterns (eg, minimal variability) are present and is a safe alternative to cesarean delivery in this setting

***Intrauterine resuscitation measures include correction of maternal hypotension (intravenous fluid and pharmacologic treatment (e.g. phenylephrine)), correction of uterine tachysystole (stop oxytocin or treat with terbutaline), maternal repositioning and oxygen administration

Fetal Malpresentation

Assess fetal presentation at 36 weeks:

Singleton	Twins
Offer external cephalic version for persistent singleton malpresentation after 37 weeks	Breech/Breech OR Cephalic/Breech w/> 20% discordance
*** If version fails, consider ECV at the time of CD<br>*** Individual studies have found a significantly greater success rate for ECV associated with the use of anesthesia	*** Perinatal outcomes for twin gestations in which the first twin is in cephalic presentation are not improved by cesarean delivery. Thus, women with either cephalic/cephalic-presenting twins or cephalic/noncephalic presenting twins should be counseled to attempt vaginal delivery<br>*** Consider MFM consult for breech extraction (provider dependent)

Suspected Fetal Macrosomia

Cesarean delivery to avoid potential birth trauma should be limited to the following:	Diabetic patient w/ US EFW >4500g<br>OR<br>Non-diabetic patient w/ US EFW >5000g	*** Patients should be counseled that estimates of fetal weight, particularly late in gestation, can be imprecise	*** Obtain tight glycemic control in patients w/ diabetes

Maternal Infection

Hx of HIV w/ viral load >1000 or unknown viral load<br>OR<br>Hx of HSV w/ active genital lesions or prodromal symptoms	*** Patients w/ Hx of HSV should be started on acyclovir 400 mg TID or valacyclovir 500mg BID from 36 weeks until delivery	*** Women with HIV should receive antiretroviral therapy during pregnancy according to currently accepted guidelines for adults<br>***Intravenous zidovudine should be given prior to CD to reduce the risk of transmisison<br>***In laboring patients (viral load <1000), clinicians may elect to use or not use intrapartum IV zidovudine

Works Cited

"ACOG Committee Opinion No. 751 Summary: Labor and Delivery Management of Women With Human Immunodeficiency Virus Infection"

"ACOG Practice Bulletin No. 82: Management of Herpes in Pregnancy."

Caughey, Aaron B., et al. "Safe Prevention of the Primary Cesarean Delivery."

" ACOG Practice Bulletin No. 116: Management of Intrapartum Fetal Heart Rate Tracings."

"ACOG Practice Bulletin No. 161: External Cephalic Version"

"ACOG Practice Bulletin No. 173: Fetal Macrosomia"

Version 1.0
